# Strategies of initiation and streamlining of antibiotic therapy in 41 French intensive care units

**DOI:** 10.1186/cc9961

**Published:** 2011-01-13

**Authors:** Philippe Montravers, Hervé Dupont, Rémy Gauzit, Benoit Veber, Jean-Pierre Bedos, Alain Lepape

**Affiliations:** 1 Département d'Anesthésie Réanimation, CHU Bichat-Claude Bernard, Assistance Publique-Hôpitaux de Paris, 46 Rue Henri Huchard, 75018, Paris, France; 2 Université Paris VII Denis Diderot, Faculté de Medecine, 16, Rue Henri Huchard, 75018, Paris, France; 3 Pôle d'Anesthésie Réanimation, CHU Hôpital Nord, Place Victor Pauchet, 80054, Amiens, France; 4 Inserm ERI 12, Université Jules Verne de Picardie, Pôle sante, 3 Rue des Louvels, 80036 Amiens, France; 5 Département d'Anesthésie Réanimation, Assistance Publique-Hôpitaux de Paris, CHU Hôtel-Dieu, 1 Place du Parvis Notre Dame, 75004, Paris, France; 6 Département d'Anesthésie Réanimation, CHU de Rouen, 1 Rue de Germont, 76031, Rouen, France; 7 Service de Reanimation Polyvalente, CH de Versailles, 177 Rue de Versailles, 78157, Le Chesnay, France; 8 Département d'Anesthésie Réanimation, CHU Lyon Sud, Hospices Civils de Lyon, Chemin du Grand Revoyet, 69310 Pierre Benite, France

## Abstract

**Introduction:**

Few studies have addressed the decision-making process of antibiotic therapy (AT) in intensive care unit (ICU) patients.

**Methods:**

In a prospective observational study, all consecutive patients admitted over a one-month period (2004) to 41 French surgical (*n *= 22) or medical/medico-surgical ICUs (*n *= 19) in 29 teaching university and 12 non-teaching hospitals were screened daily for AT until ICU discharge. We assessed the modalities of initiating AT, reasons for changes and factors associated with in ICU mortality including a specific analysis of a new AT administered on suspicion of a new infection.

**Results:**

A total of 1,043 patients (61% of the cohort) received antibiotics during their ICU stay. Thirty percent (509) of them received new AT mostly for suspected diagnosis of pneumonia (47%), bacteremia (24%), or intra-abdominal (21%) infections. New AT was prescribed on day shifts (45%) and out-of-hours (55%), mainly by a single senior physician (78%) or by a team decision (17%). This new AT was mainly started at the time of suspicion of infection (71%) and on the results of Gram-stained direct examination (21%). Susceptibility testing was performed in 261 (51%) patients with a new AT. This new AT was judged inappropriate in 58 of these 261 (22%) patients. In ICUs with written protocols for empiric AT (*n *= 25), new AT prescribed before the availability of culture results (*P *= 0.003) and out-of-hours (*P *= 0.04) was more frequently observed than in ICUs without protocols but the appropriateness of AT was not different. In multivariate analysis, the predictive factors of mortality for patients with new AT were absence of protocols for empiric AT (adjusted odds ratio (OR) = 1.64, 95% confidence interval (95%CI): 1.01 to 2.69), age ≥60 (OR = 1.97, 95% CI: 1.19 to 3.26), SAPS II score >38 (OR = 2.78, 95% CI: 1.60 to 4.84), rapidly fatal underlying diseases (OR = 2.91, 95% CI: 1.52 to 5.56), SOFA score ≥6 (OR = 4.48, 95% CI: 2.46 to 8.18).

**Conclusions:**

More than 60% of patients received AT during their ICU stay. Half of them received new AT, frequently initiated out-of-hours. In ICUs with written protocols, empiric AT was initiated more rapidly at the time of suspicion of infection and out-of-hours. These results encourage the establishment of local recommendations for empiric AT.

## Introduction

Initiation of antibiotic therapy (AT) in intensive care unit (ICU) patients is a critical issue. The importance of empiric AT covering all pathogens responsible for infections has been highlighted on many occasions [1-4]. The need for urgent AT was also emphasized in a study demonstrating a 7% increased mortality for each hour of delayed empiric AT in patients with severe sepsis and septic shock [5]. The time to the first dose of AT has been emphasized in the recommendations of the surviving sepsis campaign [6] and has become a measure of quality of care in ICU patients [7-9]. The difficulty in differentiating infectious from noninfectious etiologies in critically ill patients is also a major driver of antibiotic prescribing in ICUs leading to the development of new diagnostic tests [10]. On the other hand, the parsimonious choice of AT drugs has also been stressed to curtail the emergence of resistance and contain the cost [11,12].

Most studies addressing the issue of AT have focused on appropriateness, while few longitudinal surveillance studies have analyzed the decision-making process [1,2,4,13-15]. To more clearly understand AT current prescribing practices in ICU patients, a prospective multicenter observational study was performed to describe the modalities of initiation (frequency, timing) of AT, the reason for changes (streamline/de-escalate therapy) and identification of independent factors associated with mortality in patients receiving new AT during their ICU stay.

## Materials and methods

### Participating centers and team organization

This one-month (November 2004) prospective multicenter observational study was conducted in 41 adults surgical (*n *= 22) or medical/medico-surgical ICUs (*n *= 19) in 29 teaching university and 12 non-teaching hospitals. Participating ICUs, volunteers participating in the study, were widely distributed throughout France. These were closed units of more than six beds, non-specialized units (avoiding cardiac and neurosurgical ICUs), with a critical care specialist and microbiology laboratory on hand 24 hours a day.

Legal organization of day shifts and "out-of-hours" hours in French ICUs has been previously described [16]. Briefly, day shifts as defined by law run from Monday to Friday, 8:30 am to 6:29 pm, and Saturday from 8:30 am to 12:59 pm; the remaining period corresponds to off hours. Overall during the study period, day shifts accounted for 218 hours (30.2%) in a total of 720 hours of work.

In these units, day-shift medical teams consisted of a median of three (range, 1 to 6) senior physicians board certified in critical care medicine, a median of one (range, 0 to 3) critical care specialist in training (certified medical specialist in anesthesiology, or medical specialty), and a median of two (range, 0 to 5) residents. During out-of-hours, one critical care specialist (board certified or in training) was on call on site, either alone (in 14 ICUs) or with a medical resident.

### Study design and patients

In each center, the principal investigator was the senior critical care specialist leading the team and fully responsible for the ICU. All consecutive adult patients admitted to the ICU during the study period were eligible for enrollment. Criteria used for diagnosis, microbiologic techniques and the decision to prescribe AT were left to the physician's discretion. Ethics Committee approval for the protocol was obtained. In accordance with French law, as the study protocol was strictly observational and did not modify clinical practice, information was given to the patients and their familly but no written informed consent was obtained from our patients. Approval of the CNIL (Commission Nationale de l'Informatique et des Libertés) was obtained, ensuring that patient data were kept confidential according to French regulations. A Scientific Committee independently designed the study and reviewed all data collected.

### Clinical data

For each ICU admission, demographic characteristics, underlying diseases, severity of illness, and type of admission were recorded on a standardized report form. Severity of illness on admission was assessed using the simplified acute physiology score II (SAPS II score) [17]. Underlying diseases were classified as not ultimately fatal, ultimately fatal (death expected in <5 years) or rapidly fatal (in <1 year) according to the McCabe score [18].

**Figure 1:**
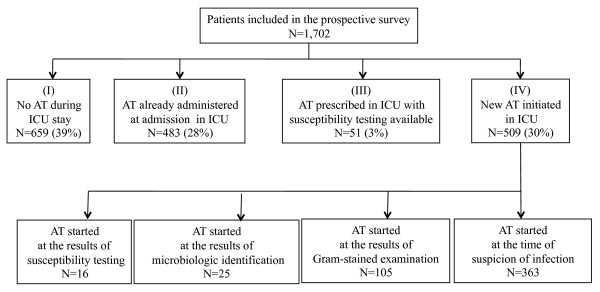
**Number and proportions of patients included in the study according to their antimicrobial therapy status**. During their intensive care unit stay: (I) Patients never receiving any antimicrobial agents; (II) patients suspected of having bacterial infection and already receiving antibiotic treatment at the time of admission; (III) patients receiving antibiotic therapy for a known infection with identification and susceptibility testing of the pathogen at the time of admission; (IV) patients receiving new antibiotic therapy for suspicion of infection during their ICU stay.

To assess the incidence of AT during the ICU stay, the patients were classified into four categories: (I) patients not receiving AT either at the time of admission, or during their ICU stay; (II) patients suspected of having bacterial infection and already receiving AT at the time of admission; (III) patients with known infection with identification and susceptibility testing of the pathogen at the time of admission on which AT was based; (IV) patients receiving new AT for a new suspicion of infection during their ICU stay (Figure 1). This last subgroup was analyzed specifically. In patients who developed several infections during their ICU stay, only the first episode of new AT was considered. A preceding seven-day course free of antibiotics was required before considering a new course of AT. Antibiotic prophylaxis was not analyzed in the current study.

### Decision-making process of AT

In each center, the presence and number of empiric AT protocols were assessed. The period of initiation of AT was defined by categorizing the week into day shifts and out-of-hours. The type of prescriber was assessed: fellow or senior physician (assistant professor, senior critical care specialist). The individual or team decision (>2 physicians) for initiation of AT was assessed. When infectious disease specialists were involved in the decision-making progress, they were considered as a part of the team. Patients with one of the following diagnoses were classified as being immunosuppressed: febrile neutropenia, splenectomized patients, cirrhosis, solid organ transplantation, steroid therapy, and HIV infection [19]. Therapeutic emergencies were defined as septic shock, hypoxemic pneumonia or multiple organ failure (MOF) [19]. The sequential organ failure assessment (SOFA) score was calculated at the time of initiation of AT [20]. The supposed source of infection was recorded.

Applied microbiologic techniques were based on the recommendations of the French Society for Microbiology [21]. Microbiologic results were recorded as part of the decision-making process for initiation or changes of AT. The definitions used for the site of infection, true pathogens, contaminants and commensals were those recommended by the French Society of Anæsthesiology and Critical Care Medicine [22]. The following timing of AT prescription was analyzed: in the absence or before microbiologic sampling; after microbiologic sampling; on the results of Gram-stained direct examination, on the results of microbiologic cultures (24 to 48 hours); on the results of susceptibility testing (Figure 1). In patients with negative cultures, the decisions were assessed 48 hours after collection of the samples when the cultures demonstrated no growth. Apart from adaptation to microbiologic results, the other reasons for antibiotic changes were recorded: clinical worsening, new site of infection, antibiotic side effect, de-escalation (withdrawing the non-pivotal antibiotic or switching to a narrow-spectrum antibiotic) and discontinuation of aminoglycosides. The quality of antibiotic prescription (dose, intervals, and so on) according to pharmacokinetic/pharmacodynamic criteria was not analyzed.

Patients treated without any microbiologic sampling of their suspected infection or having their treatment based only on microbiologic identification without susceptibility testing were considered to have a low level of microbiologic confirmation of infection. In patients undergoing susceptibility testing of their microbiologic samples, appropriateness of AT was assessed by the principal investigator at the end of the therapeutic course. In order to replicate real life conditions as much as possible, all positive microbiologic cultures were analyzed [22] but appropriateness of AT was only considered for true pathogens. Therapy was judged appropriate if, according to the susceptibility testing [21], all bacteria considered true pathogens were targeted by at least one of the drugs administered. The other cases were classified as inappropriate AT. The antibiotic selection was judged appropriate or inappropriate on the basis of the culture results obtained. Considering that severe infections encountered in ICU cases require emergency AT, the scientific committee classified the delayed introduction of AT at the time of susceptibility testing as arbitrary and inadequate AT. Fungi were excluded from the analysis of appropriateness and antifungal therapy was not considered.

### Outcome

All patients were followed from the day of admission until ICU discharge. Death during ICU stay was recorded. Links between ICU mortality and clinical features of new AT were assessed.

### Statistical analysis

Patient characteristics according to AT during their ICU stay were analyzed. Characteristics of AT were assessed and their relationships with death were determined.

Data were analyzed using Stata 9.2™ (Stata Corporation, College Station, TX, USA). We assessed that the continuous variables were normaIly distributed using the Shapiro-Wilk test. Variables were expressed as mean with standard deviation and range or numbers with proportions. Groups were compared using the Chi-square test with Yates' correction if necessary for qualitative parameters and ANOVA for quantitative data. Bonferroni correction was used for multiple comparisons. To identify factors independently associated with death, a multivariate stepwise logistic regression analysis was performed among the factors found to be significant at the 15% level in univariate analysis [23]. A backward Wald model was used. The probability to enter in the model was 0.05 and to remove 0.1. Hosmer-Lemshow goodness of fit Chi-square was assessed. The median value of the population was used as a cut-off for quantitative data. Odds-ratio (OR) and their 95% confidence intervals (95% CI) were calculated. Statistical significance was accepted at the 5% level.

## Results

### Study population

A total of 1,702 patients (Figure 1) was studied. The mean number of admissions in each unit was 42 ± 21 pts. Overall, 54 ± 30% of patients were admitted for a medical reason, 9 ± 12% following scheduled surgery, and 37 ± 25% following emergency surgery.

**Figure 2:**
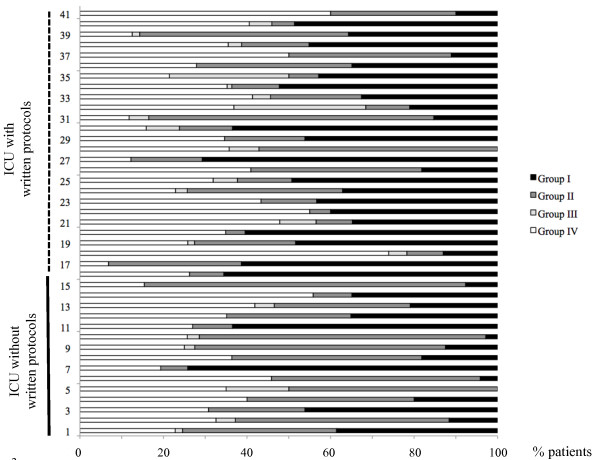
**Proportions of patients included in the study according to their antimicrobial therapy status**. During their intensive care unit stay in each ICU represented on the vertical axis. In ICUs 1 to 16 no written empiric antibiotic protocol was used while protocols were used in units 17 to 41. I) patients never receiving any antimicrobial agents; (II) patients suspected of having bacterial infection and already receiving antibiotic treatment at the time of admission; (III) patients receiving antibiotic therapy for a known infection with identification and susceptibility testing of the pathogen at the time of admission; (IV) patients receiving new antibiotic therapy for suspicion of infection during their ICU stay.

Overall, 34 ± 21% of patients did not receive any AT during their ICU stay, 29 ± 21% were already treated at the time of admission, 4 ± 7% received an AT with identification and susceptibility testing available at admission, and 34 ± 16% received new AT (Table 1). The large variation in the amount of antibiotics used by the different ICUs is illustrated by Figure 2.

**Table 1:** Main characteristics of the overall population included according to their antimicrobial therapy status

Parameters	No AT in the ICU	AT on ICU admission	AT on ICU admission and ST available	New AT in the ICU	*P*
	*N *= 659 (39%)	*N *= 483(28%)	*N *= 51(3%)	*N *= 509(30%)	
Age	54 ± 18	59 ± 17	57 ± 18	57 ± 19	<0.001
SAPS II score on admission	33 ± 21	33 ± 18	40 ± 15	41 ± 18	<0.001
Male gender	392 (59%)	323 (67%)	33 (65%)	326 (64%)	0.07
Type of admission					
scheduled surgery	145 (22%)	188 (39%)	3 (6%)	36 (7%)	
medical	367 (56%)	172 (36%)	28 (55%)	290 (57%)	<0.001
emergency surgery	147 (22%)	123 (25%)	20 (39%)	183 (36%)	
Underlying disease					
Not ultimately fatal	463 (70%)	261 (54%)	37 (73%)	329 (65%)	
Ultimately fatal	141 (21%)	175 (36%)	12 (23%)	123 (24%)	<0.001
Rapidly fatal	55 (8%)	47 (10%)	2 (10%)	57 (11%)	
AT protocols available in the ICU	380 (58%)	321 (66%)	23 (45%)	327 (64%)	<0.001
Number of empiric AT protocols available	3 ± 3	4 ± 4	2 ± 3	4 ± 4	<0.001

Data are presented as mean ± SD or as number (proportion). AT, antibiotic therapy; ICU, intensive care unit; SAPS II, simplified acute physiologic score II; ST, susceptibility testing. Underlying disease classification according to the McCabe score, see material and methods section.

### Local organization

Written protocols for empiric AT were available in 25 (61%) ICUs in accordance with national guidelines and adapted to local epidemiology, including antibiotic resistance frequencies. These protocols were defined for community-acquired infections (mainly pneumonia *n *= 19, intra-abdominal infections *n *= 19, meningitis *n *= 18) and nosocomial infections (mainly ventilator-associated pneumonia (VAP) *n *= 21, postoperative intra-abdominal infections *n *= 16, septic shock *n *= 16) with a mean of 6 ± 3 protocols per ICU. No difference was observed between teaching and non-teaching hospitals in terms of the availability (63% vs 57%, *P *= 0.72) and mean number of protocols (3 ± 3 vs 4 ± 3, *P *= 0.96). The number and availability of protocols were similar in surgical, medical and medico-surgical units.

### Decision-making process of antibiotic therapy

Among the 509 patients receiving new AT during their ICU stay, the main underlying diseases were immunosuppression (*n *= 61; 12%), respiratory and cardiovascular comorbidities (*n *= 62; 12%), cirrhosis (*n *= 31; 6%) and scored as ultimately (24%) or rapidly (11%) fatal. The mean SOFA score at the time of AT prescription was 6 ± 5. Therapeutic emergencies were reported in 42% (*n *= 215) of cases, including septic shock (*n *= 122; 24%), MOF (*n *= 47; 9%) and hypoxemic pneumonia (*n *= 1 01; 20%) with high SOFA score (11 ± 6; 13 ± 6; 9 ± 6, respectively). The most frequently suspected sites of infection were lung (*n *= 241; 47%), bacteremia (*n *= 121; 24%), and intra-abdominal (*n *= 105; 21%).

AT was initiated at the time of suspicion of infection in 363 cases (71%), based on the results of direct examination by Gram-stain in 105 cases (21%), on microbiologic cultures (*n *= 25; 5%) or susceptibility testing (*n *= 16; 3%) (Figure 1). New AT was decided on day shifts in 227 cases (45%) and out-of-hours in 282 cases (55%). New empiric AT was initiated in 213 (76%) patients out-of hours and in 150 (66%) patients on day shifts (*P *= 0.03). Treatment was based on the results of Gram-stain direct examination in 49 (17%) patients out-of-hours and in 56 (25%) cases on day shifts (*P *= 0.055), on microbiologic cultures in 14 (5%) and 11 (5%) patients, and on susceptibility testing in 6 (2%) and 10 (4%) patients, respectively. In most cases, the decision to prescribe AT was made by a single senior physician (*n *= 397, 78%, involving a senior critical care specialist (*n *= 340; 67%) or an assistant professor (*n *= 57; 11%)), and more rarely by the team (*n *= 87; 17%), or a fellow (*n *= 25; 5%).

Among the 215 patients with therapeutic emergencies, AT was initiated empirically on suspicion of infection in 152 cases (71%), in 195 (91%) at the time of the Gram-stain, on the results of microbiologic cultures in 206 cases (96%) or susceptibility tests in 214 (99.5%). Among the 121 patients suspected of bacteremia, 86 (71%) of them were treated before Gram-stain examination, 34 (28%) at the time of pathogen identification and 1 (1%) at the time of susceptibility testing. The AT decision-making process is shown in Table 2.

**Table 2:** Antimicrobial therapy characteristics according to the timing and level of microbiologic results

	AT course				
	
	No AT	AT started	Ongoing AT	AT modified	AT stopped
Clinical, radiologic or surgical suspicion of infection, *N *= 509	146 (29%)	363 (71%)	-	-	-
Gram-stained direct examination, *N *= 509	41 (8%)	105 (21%)	345 (68%)	15 (3%)	3 (1%)
Available, *N *= 204 (40%)	8	105	73	15	3
Not available, *N *= 305 (60%)	33	-	272	-	0
Microbiologic identification (24 to 48 hours), *N *= 509	23 (4%)	25 (5%)	403 (77%)	55 (11%)	3 (1%)
Available, *N *= 251 (49%)	6	25	162	55	3
Not available, *N *= 258 (51%)	17	-	241	-	0
Susceptibility testing, *N *= 509	-	16 (3%)	392 (77%)	93 (18%)	8 (1.8%)
Available, *N *= 261 (51%)	-	14	151	93	3
Not available, *N *= 248 (49%)	-	2	241	-	5

Data are presented in the patients receiving new AT (*n *= 509) and expressed as number (proportion). AT, antibiotic therapy.

No difference in the severity of the cases (assessed by SAPS II and SOFA scores) was observed according to the timing of prescription, the type of prescriber, or the time to initiation of AT.

### Role of local protocols on empiric AT

When comparing ICUs with written empiric AT protocols and those without protocols, the proportion of empiric AT among all antibiotic prescriptions was similar (33% (305 patients) of the cases per center versus 32% (204 patients), respectively) and severity scores were similar. The number of patients receiving antibiotics in units with written protocols and those without protocols was similar whenever the number of patients (12 ± 6 vs 13 ± 5 patients, *P *= 0.75) or their proportions (35 ± 19 vs 32 ± 10%, *P *= 0.56) were considered.

When compared to ICUs without protocols, a higher proportion of prescriptions was made by fellows in ICUs with written protocols (48 (14.7%) vs 12 (6.6%) in other ICUs, respectively, *P *= 0.01), AT prescriptions were more frequent at the time of suspicion of infection in ICUs with protocols (251 (76.7%) vs 112 (61.5%), respectively; *P *= 0.003) and prescription was more frequent out-of-hours in the units with a written protocol (192 (59%) vs 90 (49.5%), respectively; *P *= 0.04).

### Discontinuation and changes of empiric AT

Overall, empiric ATs were interrupted in 14 patients and modified in 163 patients following Gram-stained direct examination, microbiologic examination and susceptibility testing. Time of stopping and changes in empiric AT is summarized in Table 2.

Overall, in 346 (68%) patients no change of the new AT was made, while 191 changes were observed in 163 (31%) patients: 137 patients (27%) had one AT change, 24 (5%) two changes, and 2 (0.2%) three changes. The timing of these AT changes is presented in Table 2. Among these patients with modified AT, changes were unrelated to microbiologic reasons in 98 (19%) patients but were linked to clinical deterioration *n *= 21 (4%), to new site(s) of infection *n *= 14 (3%), to interruption of aminoglycosides *n *= 36 (7%), to adverse effects *n *= 6 (1%), or to de-escalation therapy *n *= 40 (8%).

Among the 215 patients with therapeutic emergencies, changes of AT were reported for the following reasons: 21 (10%) de-escalation, 18 (8%) interruption of aminoglycosides, 14 (6%) clinical deterioration, 4 (2%) new site(s) of infection and 2 (1%) adverse events.

### New AT in patients with a low level of microbiologic confirmation of infection

Overall 248 (49%) patients had a low level of microbiologic assessment of infection. Eighty (16%) patients (mean age 55 ± 21) received new AT without any microbiologic sampling of their suspected infection. Among these patients with a mean SAPS II score of 33 ± 15 on ICU admission, 49 (61%) were admitted for a medical diagnosis, 26 (33%) for emergency surgery and 5 (6%) for scheduled surgery. Eight (10%) were immunosuppressed, 6 (7.5%) had comorbidities and 19 (24%) had an ultimately or rapidly fatal underlying disease. Their mean SOFA score was 5 ± 5 and 10 (12.5%) had signs of therapeutic emergencies. Most of these patients were suspected of having pulmonary infection (*n *= 35, 44%) or intra-abdominal infection (*n *= 14, 18%).

In the remaining 168 cases, AT was continued with only limited microbiologic confirmation. In 59 (12%) cases, AT was prolonged and based on microbiologic identification without susceptibility testing, while 109 (21%) patients had negative cultures. Among these 59 cases with only organisms identification (SAPS II score on admission of 44 ± 17 and SOFA score of 9 ± 6 at the time of initiation of therapy), therapeutic emergencies were observed in 25 (42%) cases while therapeutic emergencies were reported in 39 (36%) of the 109 cases with negative samples (SAPS II score on admission of 38 ± 17 and SOFA score of 7 ± 6 at the time of initiation of therapy).

Overall, 51 AT changes were made among these 248 patients without susceptibility testing (including clinical deterioration in 16 cases and new site(s) of infection in 6 patients). Among the 80 patients who received a new AT without microbiologic sampling, only 11 (2%) changes were made (clinical deterioration in 4 patients, new site of infection in 2, interruption of aminoglycosides in 3, adverse effects in 2), 17 (29%) changes were made among the 59 cases who had only identification of causative organisms and 23 (21%) among the 109 patients with negative cultures.

### Appropriateness of new AT

Susceptibility testing and assessment of appropriateness of a new AT were obtained in 261 (51%) patients homogenously distributed throughout the centers. Antibiotic therapy was judged inappropriate in 58 patients (22%), involving mainly pneumonia (*n *= 26; 37.7%), bacteremia (*n *= 13; 18.8%), urinary tract (*n *= 14; 20.3%), and intra-abdominal infections (*n *= 13; 18.8%). Among the 215 cases with therapeutic emergencies, susceptibility testing and assessment of appropriateness was obtained in 126 cases (59%). Antibiotic therapy was considered appropriate in 100 cases (80%).

Patients with appropriate and inappropriate AT had similar SAPS II scores (43 ± 13 vs 42 ± 19) on admission to ICU and SOFA scores (7 ± 6 vs 7 ± 5) on initiation of AT. The clinical features at the time of initiation of AT were assessed in these 261 patients (Table 3). Some organisms initially considered as contaminants (coagulase negative staphylococci) or commensals (enterococci) turned out to be true pathogens. Consequently, the cases were classified at the end the clinical course as inappropriately treated. The reasons for additional antibiotic changes not related to susceptibility testing are shown in Table 3.

**Table 3:** Assessment of the appropriateness of antimicrobial therapy for microbiologically documented infections

Parameter	Appropriate AT	Inappropriate AT	*P*
	(*n *= 203)	(*n *= 58)	
AT protocol available in the ICU	79 (61.1%)	35 (60.3%)	0.91
Timing of new AT prescription			
Day shifts	97 (47.8%)	30 (51.7%)	0.59
Out-of-hours	106 (52.2%)	28 (48.3%)	
Category of MD prescriber			
Fellow	17 (8,4%)	7 (12.1%)	0.88
Senior physician	148 (72.9%)	41 (70.7%)	
Medical team decision	38 (18.7%)	10 (17.2%)	
Time of initiation of new AT			
Suspicion of infection	120 (59.1%)	29 (50.0%)	
Gram-stained direct examination available	65 (32.0%)	12 (20.7%)	<0.0001
Microbiologic identification available	18 (8.9%)	3 (5.2%)	
Susceptibility testing available	0	14 (24.4%)	
Change of AT			
None	107 (52.7%)	14 (24.1%)	
Gram-stained direct examination available	11 (5.4%)	4 (6.9%)	0.001
Microbiologic identification available	32 (15.8%)	11 (19.0%)	
Susceptibility testing available	53 (26.1%)	29 (50.0%)	
Number of AT changes	0.5 ± 0.6	0.9 ± 0.7	0.05
Non-microbiologic reason for AT change	38 (18.7%)	10 (17.2%)	0.79
Clinical worsening	4 (2.0%)	1 (1.7%)	
New site of infection	5 (2.5%)	4 (6.9%)	
Aminoglycoside stopped	23 (11.3%)	4 (6.9%)	
AB side effect	3 (1.5%)	1 (1.7%)	
De-escalation	26 (12.8%)	4 (6.9%)	

Data are presented among the patients receiving new AT (*n *= 509), and expressed as mean ± SD or as number (proportion). AT, antibiotic therapy; ICU, intensive care unit; MD, medical doctor.

### Links between new AT and outcome

The mean duration of ICU stay for the whole cohort was 10.8 ± 9.6 days. A 20% mortality rate (*n *= 101) was observed among the 509 patients receiving new AT with no significant differences according to gender, type of admission or type of infection (Table 4). No significant link was evidenced between mortality rate and type of institution (18% of death in university teaching hospitals compared to 23% in non-university hospitals (*P *= 0.17)) or type of ICU (17% of death in surgical ICUs, 19% in medical ICUs and 23% in medico-surgical ICUs (*P *= 0.35)). No significant link was evidenced between mortality rate and time of prescription, type of prescriber, appropriateness of AT or subsequent changes of treatment. Six the 80 patients (7.5%) who received a new AT without any microbiologic investigation finally died (including 2 of those who had changes in AT), while death was reported in 33 (30%) of the 109 cases with negative samples and 11 (19%) of the 59 patients where only the organism(s) was identified.

**Table 4:** Clinical and therapeutic characteristics of the population receiving new antibiotic treatment according to outcome

Parameter	Alive	Death during ICU stay	*P*
	(*n *= 408)	(*n *= 101)	
Age	55 ± 19	66 ± 15	0.001
Underlying diseases			
Not ultimately fatal	279 (68.4%)	50 (49.5%)	<0.0001
Ultimately fatal	95 (23.3%)	28 (27.7%)	
Rapidly fatal	34 (8.3%)	23 (22.8%)	
Immunosuppression	43 (10.3%)	18 (17.8%)	0.04
SAPS II score on admission	37 ± 15	56 ± 20	<0.0001
SOFA score at the beginning of AT	6 ± 5	12 ± 6	0.04
Severe hypoxemia	72 (17.6%)	29 (28.7%)	0.01
Septic shock	79 (19.4%)	43 (42.6%)	<0.0001
Multiple organ failure	18 (4.4%)	29 (28.7%)	<0.0001
AT protocol available	269 (65.9%)	58 (57.4%)	0.11
Number of AT protocols available	4.2 ± 3.5	3.8 ± 3.8	0.24
Category of MD prescriber			
Fellow	46 (11.2%)	10 (10%)	0.57
Senior physician	292 (71.6%)	74 (73.1%)	
Medical team decision	70 (17.2%)	17 (16.9%)	
Time of prescription of new AT			
Day shifts	185 (45.3%)	42 (41.6%)	0.49
Out-of-hours	223 (54.7%)	59 (58.4%)	
Suspicion of infection	298 (73.0%)	65 (64.4%)	0.27
Gram-stained direct examination available	77 (18.9%)	28 (27.7%)	
Microbiologic identification available	20 (4.9%)	5 (5.0%)	
Susceptibility testing available	13 (3.2%)	3 (2.9%)	
Appropriateness of new AT			
Appropriate	160 (39.2%)	43 (42.6%)	0.45
Inappropriate	50 (12.3%)	8 (7.9%)	
Not applicable	198 (48.5%)	50 (49.5%)	
Change of empiric AB			
None	286 (70.1%)	69 (68.3%)	0.65
Gram-stained direct examination available	14 (3.4%)	5 (5.0%)	
Microbiologic identification available	40 (9.8%)	13 (12.9%)	
Susceptibility testing available	68 (16.7%)	14 (13.8%)	
Number of AB changes	0.4 ± 0.6	0.4 ± 0.6	0.67

Data are presented as mean ± SD or as number (proportion). AT, antibiotic therapy; MD, medical doctor; SAPS II, simplified acute physiologic score II; SOFA, sequential organ failure assessment; Underlying diseases according to the McCabe score, see material and methods section.

Among the 509 cases, only the progress of 27 (5.3%) patients was tracked in the ICU for more than 30 days (6 deaths and 21 survivors). In the three most frequent sites of infection, mortality rates between patients receiving appropriate and inappropriate AT were not significantly different: 24/96 (25%) vs 3/26 (12%), 14/65 (22%) vs 5/15 (33%), 8/46 (17%) vs 3/13 (23%), in pneumonia, bacteremia and intra-abdominal infections, respectively. In contrast, underlying diseases and severity at the time of initiation of AT were associated with a higher mortality rate (Table 4).

Among the 98 patients who had AT changed for non-microbiologic reasons, death was observed in 8/21 (38%) patients who deteriorated clinically, in 2/14 (14%) patients who developed a new site(s) of infection, in 3/36 (8%) of those whose aminoglycosides were stopped and in 3/40 (7.5%) of those who had de-escalation therapy.

Among the 126 patients with therapeutic emergencies in whom appropriateness of AT was assessed, death was reported in 4 (15%) of the 26 patients who had inappropriate AT and 31 (31%) of the 100 patients where AT was appropriate.

Univariate and multivariate analysis assessed predictive factors of mortality in the population of patients receiving new AT (Tables 4 and 5). Hosmer-Lemshow goodness of fit Chi square was 5.06, *P *= 0.75. Among the identified risks of mortality, the absence of AT protocols was the only criterion not related to underlying disease or severity at the time of initiation of AT.

**Table 5:** Univariate and multivariate analysis of predictive factors of mortality

Parameter	OR (95%CI)	Adjusted OR (95%CI)	*P*-value
Lack of AT protocol	1.4 (0.9 to 2.2)	1.64 (1.01 to 2.69)	0.04
Age ≥60	2.6 (1.6 to 4.1)	1.97 (1.19 to 3.26)	0.008
SAPS II score on admission ≥38	4.5 (2.5 to 7.5)	2.78 (1.60 to 4.84)	<0.0001
Rapidly fatal underlying disease	3.2 (1.8 to 5.8)	2.91 (1.52 to 5.56)	0.001
SOFA score at the beginning of AT ≥6	6.2 (3.5 to 10.9)	4.48 (2.46 to 8.18)	<0.0001
Immunosuppression	1.8 (1.1 to 3.4)	---	0.26
Inappropriate AT	0.6 (0.3 to 1.3)	---	0.19
Septic shock	3.1 (1.9 to 4.9)	---	0.26
University teaching hospitals	0.7 (0.5 to 1.1)	---	0.23

Data are presented in the patients receiving new AT (*n *= 509). CI, confidence intervals; OR, odds-ratio; Rapidly fatal underlying disease (death <1 year) according to the McCabe score, see material and methods section; SAPS II, simplified acute physiology score II; SOFA, sequential organ failure assessment; AT, antibiotic therapy.

## Discussion

To our knowledge, this study represents the largest cohort addressing the AT decision-making process in ICU patients. More than 60% of patients received AT during their ICU stay and one third of them required new AT initiated out-of-hours in half of the cases.

Observational studies have their own limitations. A limited number of centers participated in the survey with heterogeneous activity and case-mix in teaching and non-teaching institutions. All microbiology laboratories followed the same guidelines published by the French Society of Microbiology [21], decreasing the heterogeneity of the management and decision-making process. The duration of the study was not sufficient to take into account seasonal changes in antibiotic prescriptions. In the study design, the decision-making process was deliberately addressed rather than considerations linked to the quality of antibiotic prescription in terms of pharmacokinetic/pharmacodynamic (pK/pD) parameters or adherence to local protocols. This issue might be relevant as the lack of correlation with microbiologically appropriate AT could be due to poor quality of the antibiotic prescription. In addition, the delay in starting new AT was not documented. This is a critical point in addressing the issue of relationship between mortality and AT and admittedly is a weakness of our study. No distinction was made between community-acquired and nosocomial infections. Finally, methodological issues could be considered as limitations. This is the case for appropriateness of antibiotic therapy assessed by local investigators, the duration of antibiotic therapy not determined and hospital mortality not assessed. Consequently, the results of this study should be interpreted cautiously, although this descriptive study can be assumed to reflect "real life" conditions.

In a single-center prospective study, Bergmans *et al*. reported that 36% of patients had at least one infection during their ICU stay and were treated for infection on 48% of all patient-days [14]. In a 15-month study in a surgical ICU using computerized patient data management systems, Hartmann *et al. *observed that 58% of the patients received AT [24]. In a single-center prospective audit, Warren *et al. *reported that 77% of admissions received at least one AT during their ICU stay [13]. In this paper, 17% of AT were initiated prior to ICU admission and 45% of patients received antibiotics for suspected or proven sepsis [13]. In a study performed in 23 Swedish ICUs over a two-week period, the median proportion of patients receiving antibiotics was 74% (range 24 to 93%); 64% of all prescriptions corresponded to empiric AT with only minor differences between units [15]. In a Turkish six-month single-center study, AT was prescribed in 61% of all admissions and empiric therapy accounted for 46% of cases [25]. In the EPIC II study, 9,084 (71%) of 13,796 adult patients in 1,265 ICUs from 75 countries were receiving antibiotics in this point prevalence study [26].

In more than 70% of our patients receiving AT, treatment was initiated before the results of Gram-stained direct examination and at the time of direct examination in more than 90% of these patients. In a prospective Spanish multicenter study in severe sepsis, the authors observed that 66% of patients received broad-spectrum antibiotics during the first six hours after presentation [7]. In a recent French multicenter study performed over six months in 2006, the authors reported that antibiotic therapy was administered within the first three and six hours following the diagnosis of severe sepsis or septic shock in 46% and 61% of patients, respectively [27]. Other studies addressing the delay of AT have reported similar observations of treatments administered within the first three to six hours in 60 to 86% of patients [8 ,9].

Heterogeneity of practice with regard to microbiological sampling was not a surprise. In a previous observational study addressing the treatment of postoperative pneumonia, we reported that 14% of the patients received empiric AT without pulmonary samples having been taken [28]. While half of these of patients were hospitalized in ICU at the time of diagnosis only 6% of them developed ventilator associated pneumonia. These were mainly the less severe cases. The second major source for early treatment without sampling was the absence of round-the-clock microbiological laboratory facilities. This was not the case in our study where all ICUs had direct access to the laboratory.

To our knowledge, very few studies have evaluated whether initiation of AT during out-of-hours modifies the appropriateness of antimicrobial therapy. We hypothesized that out-of-hours could be associated with a lower proportion of appropriate AT, especially among the least experienced ICU physicians. Interestingly, no such differences were observed considering appropriateness of AT or outcome. This point was also not observed in centers without written guidelines. However, the proportion of fellow prescribers is too small to draw any conclusions. Inexperienced physicians may have a tendency to start a broad spectrum AT regime and this perhaps explains why no correlation between level of training and appropriateness was found. Local protocols and guidelines might play a protective role in that more antimicrobials are prescribed more securely by on-call doctors and more often at the beginning of infection probably ensuring earlier initiation of treatment.

Defining appropriateness of AT is a major challenge. This issue can be assessed in many ways. Gyssens *et al. *have developed an interesting algorithm to assess comprehensively the quality of antibiotic prescriptions [29]. Basing it only on a match between the antibiotic given and the results of susceptibility testing is the commonest approach used in the literature and makes sense with regard to patient outcome in severe infections. However, this mode of prescribing is perhaps short-sighted. Even if broad spectrum AT is much more likely to be "appropriate" than limited spectrum AT in the circumstances, the ecologic issues and risks of emergence of resistance with such a policy are major concerns.

De-escalation following AT appears to vary considerably, depending on the initial diagnosis from 23% of all antibiotic prescriptions [13] to 64% in patients with septic shock [4]. However, in many instances, no microbiologic confirmation is obtained or susceptibility testing is not available, which raises the issue of de-escalation. This has been frequently demonstrated where there is suspicion of pulmonary infection, as many noninfectious processes present with lung infiltrates and fever, falsely attributed to pneumonia [30,31]. In ventilator associated pneumonia, as many as 30% of clinically suspected cases are not confirmed microbiologically [32], while in surgical ICU patients, Singh *et al. *[33] reported that only 30% of pulmonary infiltrates were the result of pneumonia. De-escalation is, therefore, problematic in these cases [34] and should be considered cautiously especially in therapeutic emergencies. In the absence of confirmation of infection (for example, negatives cultures in a patient already receiving AT), de-escalation is difficult and the appropriateness can only be evaluated by compliance to the protocols.

The proportions of appropriate AT in ICU patients are usually situated in the range of 70 to 80% of cases [1,2,4,9] and up to 89% in some specific diagnoses [4]. The proportion of documented septic episodes was only slightly greater than 50% in our study and evaluation of appropriateness was based on documented cases. In the study by Kumar [5], appropriateness was also evaluated in non-documented infections by comparing the treatment to local written guidelines.

The absence of a significant link between mortality and appropriateness of AT is somewhat surprising and appears to contradict one the findings of the study: the lack of treatment protocols was an independent risk factor for increased mortality. An explanation for this paradox could be linked to the heterogeneity of the study population involving an insufficient number of patients to reach a significant threshold to observe an effect of inappropriateness. Previous studies demonstrating the importance of appropriateness from AT usually used larger cohorts of patients [1,2,5,35] or analyzed selected populations with a single disease [3,4,35,36]. The role played by young prescribers might also be considered. Inexperienced physicians as mentioned earlier may rather have a tendency to start a broad therapy regime which might explain why no correlation between level of training and appropriateness was found. In addition, the possibility of misdiagnosis cannot be excluded, since appropriateness of AT did not include this criteria. Information about delays in initiating AT would also have been of value in explaining our observations.

Many reports have shown that the use of antimicrobial guidelines was associated with improved appropriate antibiotic use, decreased duration of AT, reduced antibiotic costs and could decrease mortality, as observed in hospital-acquired pneumonia [37,38]. The use of guidelines could be a surrogate marker for a better quality of care in general in the ICUs, thereby explaining the link between availability of guidelines and prognosis. This point was clearly emphasized in the studies assessing the effects of implementation of the guidelines of the surviving sepsis campaign [27,39]. In view of the limitations of our work, the observations about the use of guidelines and prognosis should be considered cautiously. However, it is of interest to notice that among the risk factors of mortality identified in our patients, the absence of AT protocols was the only parameter that could be easily modified, as the other risk factors were linked to underlying disease or severity at the time of AT.

In view of our data, almost 20% of antibiotic prescriptions might be unnecessary in patients with suspected infection. In this setting, antimicrobial stewardship programs might be useful [40]. Developing protocols in association with infection control measures could be considered a first step of improving antibiotic use.

Of all indications for AT, the respiratory tract is by far the most important site of infection accounting for 47% of all infections in our cohort, and almost half of these cases corresponded to severe hypoxemic pneumonia. These pulmonary infections are the most frequent reason for AT in ICUs, reported in 43 to 51% of cases [13,14], and up to 60% in a context of septic shock [4]). The frequency and severity of these cases might justify large-scale diffusion of local protocols concerning this specific issue.

## Conclusions

In view of the limited number of publications on this topic, our results should be of interest to clinicians in the field. Our observations show that more than half of the patients admitted in ICU received antibiotics during their stay, half of them on an empiric basis. Half of these treatments may not be justified on the basis of negative microbiologic cultures. In ICUs with written protocols for empiric AT, treatments might be initiated more rapidly at the time of suspicion of infection and out-of-hours. These observations should encourage the establishment of local AT protocols to initiate AT without delay and to stop the abuse of AT. Since pulmonary infections are the most frequent type of infection and as septic shock and MOF are the most life-threatening infections, local guidelines should start by addressing these issues.

## Key messages

• More than half of all critical care patients receive antibiotic therapy during their ICU stay.

• Half of the antibiotic treatments administered in ICUs are initiated on an empiric basis.

• Empiric antibiotic prescriptions are more frequent at the time of suspicion of infection in ICUs with written protocols

• Empiric antibiotic prescriptions are more frequent out-of-hours in the units with a written protocol

• De-escalation therapy and minimizing the abuse of antibiotic therapies should be discussed comprehensively and accurately.

## Abbreviations

AT: antibiotic therapy; CNIL: (Commission Nationale de l'Informatique et des Libertés); CI: confidence interval; ICU: intensive care unit; MD: medical doctor; MOF: multiple organ failure; OR: odds-ratio; pK/pD: pharmacokinetic/pharmacodynamic; SAPS II: simplified acute physiologic score II; SOFA: sequential organ failure assessment; VS: versus.

## Competing interests

The authors declare that they have no competing interests.

## Authors' contributions

All authors participated equally in the work and were involved in the design of the study, in the statistical analysis, and drafting of the manuscript. All authors had full control of primary data and agreed to allow the journal to review the data if requested. All authors read and approved the final manuscript.
